# The importance of health behaviours in childhood for the development of internalizing disorders during adolescence

**DOI:** 10.1186/s40359-017-0208-x

**Published:** 2017-12-12

**Authors:** Xiu Yun Wu, Sara F. L. Kirk, Arto Ohinmaa, Paul J. Veugelers

**Affiliations:** 1 Healthy Populations Institute, Dalhousie University, Halifax, NS Canada; 2 IWK Health Centre, Halifax, NS Canada; 3 School of Public Health, University of Alberta, Edmonton, AB T6G 1C9 Canada

**Keywords:** Children, Adolescents, Diet quality, Physical activity, Sedentary behaviour, Internalizing disorders, Health behaviours

## Abstract

**Background:**

Poor mental health constitutes a considerable global public health burden with approximately half of all cases of poor mental health having their onset before the age of 14 years. The identification of modifiable risk factors early in life is therefore essential to prevention, however, there are presently very few longitudinal studies on health behaviours for mental health to inform public health decision makers and to justify preventive action. We examined the importance of diet quality, physical activity (PA) and sedentary behaviours in childhood for internalizing disorder throughout adolescence.

**Methods:**

We linked data from a population-based lifestyle survey among 10 and 11 year old grade five students in the Canadian province of Nova Scotia with physician diagnoses of internalizing disorders from administrative health records. We applied negative binomial regressions to examine the associations of health behaviours with the number of health care provider contacts with a diagnosis of internalizing disorder.

**Results:**

Of the 4875 students, 23.9% had one or more diagnoses for internalizing disorder between the age of 10 or 11 years and 18 years. The number of health care provider contacts with a diagnosis of internalizing disorder was statistically significant higher among students with less variety in their diets, and among students who reported less PA and more time using computers and video games. The number of health care provider contacts was also higher for girls, and for students with low self-esteem and from low-income households.

**Conclusions:**

These findings suggest that diets and active lifestyles in childhood affect mental health during adolescence, and imply that succxessful health promotion programs targeting children’s diets and activity will contribute to the prevention of mental health disorders in addition to the prevention of chronic diseases later in life.

**Electronic supplementary material:**

The online version of this article (10.1186/s40359-017-0208-x) contains supplementary material, which is available to authorized users.

## Background

Poor mental health constitutes a considerable global public health burden with approximately one in four people experiencing one or more episodes of poor mental health throughout their lifetime [1, 2]. Approximately half of all cases of poor mental health have their onset before the age of 14 years [3]. Individuals experiencing poor mental health in childhood and adolescence are more likely to develop more severe forms of mental health challenges and to engage in risky behaviours like substance use, or attempt suicide in adulthood [4, 5].

Various cross-sectional studies among children and adolescents have reported associations of diet [6, 7], physical activity (PA) [8–12] and sedentary behaviour (SB) [13–16] and mental health status, characterized as internalizing disorders (including depression, anxiety, distress, mood disorder). However, there are few longitudinal studies that have examined the importance of these health behaviours in childhood for mental health later in life [17–25], and those that exist have produced incomplete or inconsistent findings. We have identified only two prospective studies that reported a significant relationship between low diet quality and poor mental health in adolescents [17, 18], whereas others reported little or no relationship [19]. While several prospective studies have indicated positive effects of physical activity on future mental health in adolescents and young adulthood [20–23], other studies have not found such an effect [24–26]. Relative to childhood PA for mental health, the association of sedentary behaviour with mental health among children and youth has been less investigated [13] and the evidence of sedentary behaviour as a risk factor for development of poor mental health in youth is scarce [13, 14]. Some cross-sectional studies have demonstrated an adverse effect of sedentary behaviour on mental health in children and adolescents [9, 10, 15, 16]. Nevertheless, population-based longitudinal studies among youth are lacking for the effect of sedentary behaviour on the mental health problem of internalizing disorders, in which the potential confounding influence of socioeconomic factors as well as other health behaviours (e.g., PA, diet quality) is considered. The limitations of existing studies on the aforementioned health behaviours for mental health include use of cross-sectional designs, few prospective studies with limited follow up, or not accounting for other important indicators and diverse socioeconomic confounders [6, 8, 13]. For example, most of the prior studies on health behaviours and mental health in youth have examined a single or two behaviours (e.g., diet or PA or sedentary behaviours), rather than considering multiple health behaviours and socioeconomic confounders simultaneously [9–11, 13]. In addition, very few studies have included indicators of specific aspects of self-esteem in childhood for future mental health in adolescence [27]. Moreover, most studies have relied on self-reported measures of mental health disorders rather than on a physician diagnosis as a manifestation of objective and clinically significant mental health issues. More longitudinal studies are needed to better understand influences of diet quality, PA and sedentary behaviours on mental health disorders.

Previously we reported that diet quality, in terms of more variety of food items in the diet, was associated with a lower risk of physician diagnosed internalizing disorders among a population-based sample of children over three years of follow up [19]. The present study extends this work by examining several health behaviours, diet quality, PA and sedentary behaviours, as well as self-esteem in relation to physician diagnosed internalizing disorders during 8 years of follow up, with the aim to better inform public health decision makers on intervention programs to improve mental health outcomes among children and youth. Based on established literature we identified diet quality, PA and sedentary behaviour as potentially relevant health behaviours, and sociodemographics as potential confounders [7, 8, 13, 17, 24, 27]. The purpose of the study was to investigate the effect of these health behaviours in childhood on the number of health care provider contacts with a primary diagnosis of internalizing disorders in adolescence. The number of health care provider contacts with a primary diagnosis of internalizing disorders given by a physician or a medical specialist was derived from administrative health data in the province of Nova Scotia. Administrative health data has been widely used in mental health epidemiologic and health services research, and has been demonstrated feasible and valid in identifying a mental disorder [28, 29].

## Methods

This examination of the relationship between health behaviours and internalizing disorders used behavioural information taken from a survey, the 2003 Children’s Lifestyle and School Performance Study (CLASS) [30], linked with physician diagnoses obtained from administrative health records.

### The survey

The CLASS study was a population-based survey among grade five students, who were primarily 10 and 11 years old at the time of survey administration, and their parents in the province of Nova Scotia, Canada. The study examined the importance of health behaviours and academic performance. Of all 291 provincial public schools with grade five students, 282 schools participated in the study. The average participation rate was 51.1% per school. Of the 5517 grade five students who received parental consent to participate in the study, 5200 students completed the surveys [30].

The CLASS study consisted of a home survey that was completed by parents, a student survey with questions on PA, sedentary activities and self-esteem, and a Canadianized version of the Harvard Youth/Adolescent Food Frequency Questionnaire (YAQ) [31] administered in the schools by study assistants. Study assistants also measured standing height of the students to the nearest 0.1 cm and body weight to the nearest 0.1 kg on calibrated digital scales. The home survey collected information on children’ place of residency, gender, household income, and highest level of parental education. In addition, participating parents provided the Nova Scotia health insurance number for their child and consent to allow for future data linkage with administrative health databases.

### Administrative health data

The administrative health data were derived from the Medical Services Insurance (MSI) database and the Canadian Institute for Health Information Discharge Abstract Database (CIHI DAD). The MSI database is administered by Medavie Blue Cross for the province of Nova Scotia and contains records for each insured health service rendered by a physician (including emergency room visits) and paid for by the Nova Scotia provincial health care system. The CIHI DAD contains a comprehensive administrative transcription of each admission to a Nova Scotia hospital facility. Both of these databases contain individual patient-level information including patient demographic characteristics, visiting physicians, diagnoses and procedures received, service transfers while in hospital, and specialty services received. Data were available from 1992 (when the grade five students who participated in 2003 were born) to 2011 (when participating students turned 18 years of age).

Of the 5200 students who completed the survey, 4875 (94%) provided valid health card numbers and were successfully linked with the administrative health data.

### Health behaviours

The exposures of interest were diet quality, PA and sedentary behaviours. On the basis of students’ nutrient intake and dietary information from the YAQ [31] and Canadian Nutrient Files [32], we calculated intake of nutrients and daily energy intake, number of daily servings of fruits and vegetables. We then calculated the Diet Quality Index (DQI) based on the composite measure, DQI-international (DQI-I) [33]. The DQI-I scores ranges between 0 and 100, with higher scores indicating a better diet quality. The DQI-I constitutes four components: variety, adequacy, moderation and overall balance of the diet. DQI-I variety evaluates the extent to which food intake comes from diverse sources within and between food groups. DQI-I adequacy examines whether the intake of foods is adequate to meet the requirement for a healthy diet. DQI-I moderation assesses intakes of foods and nutrients (i.e. fat intake) that need restriction due to their associations with chronic diseases. DQI-I balance examines the proportion of intakes from different energy sources. The DQI-I balance was categorized into two groups: ‘poor’ and ‘good’ balance. All other diet quality indicators were divided into tertiles, with higher tertiles indicating better diet quality.

The CLASS survey included questions from the National Longitudinal Survey for Children and Youth [34] on playing sports or physical activities with and without a coach. The question was reported as weekly engagement in the physical activities: Never, 1 to 3 times/week, ≥4 times/week. Sedentary behaviour was captured through questions on daily number of hours spent on playing computers or video games, and on watching television, with less than 1 h/day, 1–2 h/day, 2–4 h/day, and ≥5 h/day as response categories.

### Internalizing disorders

Participants were considered to have a mental disorder if they received one or more diagnoses of internalizing disorders according to the International Classification of Diseases, Ninth Revision, Clinical Modification (ICD-9-CM) or Tenth Revision, (ICD-10-CA). The ICD-9 and ICD-10 codes for internalizing disorders in this study are shown in Table 1. All diagnoses of internalizing disorders between 2003 when children were 10 to 11 years and 2011 when the participants turned 18 years were considered.

**Table 1:** ICD-9 and ICD-10 codes identifying diagnosis of internalizing disorder

Internalizing disorder	ICD-9 code	ICD-10 code
Depressive episode, recurrent depressive disorder, recurrent/persistent or unspecified mood disorder (excluding bipolar), neurotic disorder, general anxiety disorder, reaction to stress, adjustment reaction, emotional disorders	296, 296.2, 296.3, 296.9, 300, 308, 309, 311, 313	F32, F33, F34, F38, F39, F40, F41, F42, F43, F48, F92, F93

### Confounders

We considered gender, household income, parental educational attainment, residential location, body weight status, and self-esteem as potential confounders in the relationship between health behaviours and internalizing disorders. We considered household income at 4 levels*: $0 to $20,000; $20,001–$40,000; $40,001- $60,000; >$60,000,* parental education attainment as *secondary school or less; college,* and *university or above.* Residential location was classified as *urban and rural area*. We applied the age- and gender-specific body mass index cut-off points for children established by the International Obesity Task Force [35] to categorize body weights as *normal weight, overweight and obesity*. Total energy intake was also adjusted for as per established recommendations for analysing food frequency data [36].

The CLASS survey included 11 questions related to self-esteem. Response options for each were *‘Never or almost Never’, ‘Sometimes’ and ‘Often or almost Always’* [37]. By means of principal component analysis (PCA), we reduced the 11 questions to four components: self-perception, externalizing problems, internalizing problems and social-perception [38]. The predicted self-esteem scores for each of these four components were considered as confounders as self-esteem is strongly associated with mental health and potentially associated with health behaviours [27, 38].

### Statistical analysis

Descriptive analysis was used to present the frequency distribution of socio-demographic characteristics and health behaviours of children and the percentage with internalizing disorders in adolescence. The Chi-square test was applied to assess differences in weighted estimates for internalizing disorders between groups by socio-demographic and health behaviours. To examine the association of diet quality, PA, sedentary behaviour with internalizing disorders, we applied univariable and multivariable negative binomial regression models (NBM) using the number of health care provider contacts for internalizing disorders as the outcome. NBM is preferred over Poisson regressions when the outcome data is over-dispersed [39] which was the case in the present study. The multivariable NBM was adjusted for the confounding influence of students’ gender, residential location, household income, parental education, body weight status, energy intake and self-esteem. We also performed univariable and multivariable logistic regressions to examine the effect of the health behaviours on the diagnosis of internalizing disorders, where a binary outcome variable was used indicating whether the students received a diagnosis of an internalizing disorder during the follow up. Missing values for household income (23.2%), parental education (7.2%) and body weight status (20.2%) were considered as separate covariate categories in the regression models, and the estimates for these categories were not presented. As response rates in residential areas with lower household income were slightly lower than the average, we weighted the analyses using response weights such that the estimates represent the population of grade five students in the province of Nova Scotia [40]. The statistical tests for significance were set at *p* < 0.05. We used the statistical software of STATA 13.1 (Stata Corp., College Station, TX, USA) for the statistical analysis.

## Results

Over the course of approximately eight years when students matured from age 10 or 11 to age 18 years, 1164 (23.9%) had one or more health care provider contacts with a diagnosis of internalizing disorder following the CLASS survey (Table 2). Girls had a higher proportion (28.1%) of health care provider contacts for internalizing disorders than boys (19.9%). The proportion of students with a diagnosis of internalizing disorders was higher among students who reported lower PA levels and students from low income households, and students attending schools in urban areas.

**Table 2:** Socio-demographic characteristics and percentage (%) with an internalizing disorder among participants of the Children’s Lifestyle and School Performance Study, Nova Scotia, Canada

Variable	Percentage	Percentage with internalizing disorder
Physical activity without coach		*P = 0.103*
Never	9.2	28.1
1 to 3 times/week	34.0	23.7
≥ 4 times /week	56.8	23.4
Physical activity with coach		*P = 0.003*
Never	37.5	26.5
1 to 3 times/week	44.8	23.0
≥ 4 times /week	17.8	20.8
Use of computers or playing video games		*P = 0.079*
Less than 1 h/day	42.7	22.1
1–2 h/day	38.7	25.5
3–4 h/day	12.3	25.4
≥ 5 h/day	6.3	23.1
Watching TV		*P = 0.449*
Less than 1 h/day	14.6	25.8
1–2 h/day	40.2	22.6
3–4 h/day	29.2	24.7
≥ 5 h/day	16.0	24.5
Body weight status		*P = 0.635*
Normal weight	67.2	24.9
Overweight	23.0	24.4
Obese	9.8	22.6
Household income		*P < 0.001*
< $20,000	11.9	33.7
$20,001–$40,000	22.6	26.2
$40,001–$60,000	26.4	24.2
> $60,000	39.1	19.9
Parental education		*P = 0.027*
Secondary school or less	30.5	26.2
College	37.6	23.9
University or above	31.9	21.8
Residence		*P = 0.004*
Rural	32.7	21.5
Urban	67.3	25.3
Gender		*P < 0.001*
Boys	49.2	19.9
Girls	50.8	28.1

All estimates were weighted to represent estimates for grade five students in Nova Scotia. The Chi-square test was used to obtain the *P*-values in the table

Of the 1164 students who had one or more health care encounters for internalizing disorders, 52.3%, 19.3%, 9.6% and 18.8% of them had one, two, three and four or more service encounters, respectively between age 10 or 11 and age 18 years. Table 3 presents the univariable and multivariable adjusted incidence rate ratios (IRR) and 95% confidence intervals (CIs) for the association between health behaviours and number of health care encounters with diagnosis of internalizing disorders. The multivariable analyses presented under Model 1 are adjusted for the confounding potential of characteristics listed in Table 3 with the exception of self-esteem, and those under Model 2 are adjusted for all characteristics in the table including self-esteem. Diet variety, physical activity without a coach, use of computers and playing video games were found to be associated with the number of service encounters for internalizing disorders in a statistical significant manner. Relative to children in the lowest tertile, children in the middle tertile for diet variety had 25% lower health care provider visits with a diagnosis of internalizing disorders (Table 3, Model 1). Children who played sports or undertook PA without a coach 1 to 3 times a week had a lower rate (IRR = 0.69, 95% CI: 0.51, 0.93) of receiving health care for internalizing disorders than children who never played sports or undertook PA without a coach. More time spent on using computers or playing video games was associated with a higher number of health care provider contacts for a diagnosis of internalizing disorders. These risk estimates remained largely the same when further adjusted for self-esteem (Table 3, Model 2). Children reporting poor self-esteem for the internalizing sub-scale (IRR = 1.26, 95% CI: 1.05, 1.51) and for the social-perception sub-scale (IRR = 1.39, 95% CI: 1.15, 1.69) were more likely to receive health care related to internalizing disorders. The association between body weight status and the diagnosis for internalizing disorders (Table 2) and the number of visits receiving health services for internalizing disorders (Table 3) was not statistically significant (*P* > 0.05).

**Table 3:** Associations of health behaviours in childhood with number of health care provider contacts with a primary diagnosis of an internalizing disorder in adolescence among participants of the Children’s Lifestyle and School Performance Study, Nova Scotia, Canada

Variable	Univariable Model	Multivariable Model 1	Multivariable Model 2
	IRR (95% CI)	IRR (95% CI)	IRR (95% CI)
DQI-I variety			
Lowest tertile	1.0	1.0	1.0
Middle tertile	0.81 (0.66, 1.01)	**0.75 (0.58, 0.98)**	**0.77 (0.60, 0.99)**
Highest tertile	0.93 (0.72, 1.20)	0.84 (0.60, 1.19)	0.84 (0.59, 1.18)
DQI-I moderation			
Lowest tertile	1.0	1.0	1.0
Middle tertile	1.02 (0.82, 1.28)	0.98 (0.72, 1.32)	0.97 (0.71, 1.34)
Highest tertile	1.15 (0.89, 1.48)	1.10 (0.77, 1.57)	1.10 (0.76, 1.58)
DQI-I adequacy			
Lowest tertile	1.0	1.0	1.0
Middle tertile	1.04 (0.82, 1.31)	1.22 (0.90, 1.65)	1.31 (0.95, 1.80)
Highest tertile	1.04 (0.81, 1.33)	1.19 (0.79, 1.78)	1.26 (0.83, 1.91)
DQI-I balance			
Poor balance (score < 1)	1.0	1.0	1.0
Good balance (score ≥ 1)	1.04 (0.86, 1.27)	0.94 (0.76, 1.17)	0.91 (0.73, 1.13)
DQI-I overall			
Lowest tertile	1.0	1.0	1.0
Middle tertile	0.89 (0.70, 1.14)	0.92 (0.73, 1.17)	0.97 (0.77, 1.23)
Highest tertile	1.02 (0.81, 1.29)	1.17 (0.79, 1.73)	1.21 (0.83, 1.76)
Physical activity without coach			
Never	1.0	1.0	1.0
1 to 3 times/week	**0.71 (0.51, 1.00)**	**0.69 (0.51, 0.93)**	**0.70 (0.51, 0.96)**
≥ 4 times/week	0.78 (0.56, 1.07)	0.81 (0.60, 1.09)	0.83 (0.61, 1.13)
Physical activity with coach			
Never	1.0	1.0	1.0
1 to 3 times/week	**0.79 (0.64, 0.98)**	0.87 (0.71, 1.07)	0.87 (0.71, 1.07)
≥ 4 times /week	**0.62 (0.46, 0.85)**	0.76 (0.57, 1.02)	0.79 (0.58, 1.07)
Use of computers or playing video games			
Less than 1 h/day	1.0	1.0	1.0
1–2 h/day	**1.40 (1.12, 1.74)**	**1.57 (1.27, 1.93)**	**1.56 (1.26, 1.93)**
3–4 h/day	1.15 (0.87, 1.52)	**1.49 (1.12, 1.98)**	**1.42 (1.06, 1.91)**
≥ 5 h/day	1.18 (0.83, 1.68)	**1.78 (1.19, 2.67)**	**1.67 (1.10, 2.54)**
Watching TV			
Less than 1 h/day	1.0	1.0	1.0
1–2 h/day	0.97 (0.74, 1.27)	0.97 (0.74, 1.27)	0.98 (0.74, 1.29)
3–4 h/day	1.04 (0.79, 1.36)	0.93 (0.71, 1.22)	0.93 (0.71, 1.23)
≥ 5 h/day	0.85 (0.65, 1.12)	0.79 (0.58, 1.07)	0.77 (0.56, 1.06)
Body weight status			
Normal weight	1.0	1.0	1.0
Overweight	1.08 (0.81, 1.44)	1.07 (0.84, 1.36)	1.05 (0.83, 1.32)
Obese	1.13 (0.73, 1.75)	0.93 (0.68, 1.28)	0.85 (0.62, 1.18)
Gender			
Boys	1.0	1.0	1.0
Girls	**1.62 (1.32, 1.98)**	**1.64 (1.36, 1.98)**	**1.61 (1.33, 1.96)**
Household income			
< $20,000	1.0	1.0	1.0
$20,001–$40,000	0.74 (0.54, 1.01)	0.76 (0.57, 1.02)	0.82 (0.61, 1.11)
$40,001–$60,000	0.81 (0.57, 1.15)	0.81 (0.60, 1.09)	0.84 (0.62, 1.14)
> $60,000	**0.57 (0.41, 0.78)**	**0.54 (0.40, 0.73)**	**0.59 (0.43, 0.79)**
Parental education			
Secondary school or less	1.0	1.0	1.0
College	0.80 (0.63, 1.02)	0.87 (0.70, 1.08)	0.84 (0.68, 1.05)
University or above	0.81 (0.63, 1.05)	0.94 (0.73, 1.20)	0.98 (0.76, 1.27)
Residence			
Rural	1.0	1.0	1.0
Urban	**1.44 (1.17, 1.76)**	**1.65 (1.37, 1.99)**	**1.73 (1.43, 2.1)**
Self-esteem (Low vs. high self-esteem)			
Self-perception	**1.24 (1.01, 1.52)**		1.19 (0.98, 1.43)
Externalizing problems	0.95 (0.76, 1.19)		1.02 (0.81, 1.27)
Internalizing problems	**1.29 (1.05, 1.59)**		**1.26 (1.05, 1.51)**
Social-perception	**1.35 (1.10, 1.67)**		**1.39 (1.15, 1.69)**

*IRR* Incidence rate ratio, *95% CI* 95% confidence interval; Model 1: IRR’s are mutually adjusted for all variables in the table and for energy intake but not for the self-esteem variables; Model 2: IRR’s are mutually adjusted for all variables in the table, including the self-esteem variables, and for energy intake. Estimates are weighted to represent grade five students in Nova Scotia. Bold values for IRRs and 95% CIs indicate statistical significance (*p* < 0.05)

The risk estimates for diagnoses of internalizing disorders after adjustment for diagnoses of internalizing disorders prior to the survey in 2003 were similar to those presented in Table 3. In addition, logistic regression analyses providing risk estimates for health behaviours with respect to the first diagnosis of internalizing disorders were similar, though less pronounced, as those presented in Table 3. The results of the logistic regression analyses are included in the Additional file 1: Table S1.

## Discussion

This study reveals that low dietary variety, physical inactivity and sedentary behaviour, as measured by increased time spent on use of computers and playing video games in childhood, were associated with a greater number of health care encounters with diagnoses of internalizing disorders during adolescence. As these associations are independent of socio-demographic factors, body weight and self-esteem in childhood, they suggest these health behaviours are associated with the development of internalizing disorders during adolescence.

We observed that children with diets comprising a good variety of food items were less likely to develop internalizing disorders throughout adolescence, consistent with our earlier work showing this for a three-year period of follow up [19]. Other research has shown that adults who consume a greater variety of foods are also more likely to consume nutrient-dense foods to achieve a balanced diet [41]. Exposing children and youth to a wide variety of food sources may therefore help them meet their nutritional needs, thereby reducing the likelihood of developing internalizing disorders. While a prospective study in Australian adolescents aged 11–18 years reported an association between poor diet quality and mental health problems during 2-years of follow-up [17], our observations suggest that inadequate dietary variety may be the more salient aspect of diet quality that contributes to the development of internalizing disorders.

Benefits of physical activity for mental health among children and adolescents have been well documented in cross-sectional studies [8]. The existing longitudinal studies on the effect of physical activity on mental health in adolescents and young adults have been less consistent [20–26, 42, 43]. A longitudinal study among German adolescents and young adults reported that physical activity was associated with a lower incidence of mental disorders over a 4-year follow-up [42]. For US adolescents followed for 2 years, Motl et al. reported that a change in frequency of physical activity was related to an inverse change in depressive symptoms [43]. A more recent study examining the association between leisure time PA in adolescents and mental health in early adulthood, found that a low level of leisure time PA in adolescent girls was related to poor mental health at 20/21 years old [20]. The above findings are consistent with our observation that lower rates of physical activity without a coach are associated with the development of internalizing disorders over a period of eight years. We also observed that lower rates of physical activity with a coach was associated with internalizing disorders but this association appeared not to be statistically significant when confounders were considered (Table 3). In contrast, several other prospective studies observed no association between physical activity and later depressive symptoms [24–26, 44].

We had examined the effect of two different indicators of sedentary behaviour on internalizing disorders, one being the use of computers or playing video games, and the other watching TV. Our observation that children who used computers and video games more frequently were more likely to develop internalizing disorders seems consistent with studies reporting that playing computers and video games is related to an elevated risk of mental health problems among children and adolescents such as depression, anxiety, and emotional problems [15, 45, 46]. Our observation that TV watching was not associated with internalizing disorders is also consistent with findings from some studies [15, 45, 47], though other studies did report an association with depression, one of the common internalizing disorders [48, 49]. Differences across studies may arise from different assessment methods, differences in mental health definitions, differences in duration of follow up, and whether other health behaviours were considered in the statistical analyses. The present study contributes to the literature by demonstrating a prospective association of use of computers and video games with internalizing disorders during adolescence, independent of physical activity levels, diet quality, socio-demographic characteristics and self-esteem. This is in agreement with a study showing that screen time in children is related to psychological problems irrespective of physical activity [50].

The effects of socio-demographic characteristics of students on internalizing disorders are in line with those of previous studies [51–53]. Girls used more health care services for internalizing disorders (IRR = 1.61, 95% CI: 1.33, 1.96) than boys during the follow up period. Higher levels of household income were strongly associated with lower health care contacts with physicians for internalizing disorders (Table 3). The results highlight the importance of targeting mental health promotion programs towards girls and those children among socioeconomically disadvantaged communities and families.

Policies and programs promoting healthy eating and active living among children aim to reduce excess body weight and prevent the development of chronic diseases later in life. The findings of the present study suggest that such policies and programs may also reduce the burden of internalizing disorders in adolescence and in adulthood. Where programs promote healthy eating, active living and self-esteem simultaneously, the benefits for mental health are expectedly higher as the present study revealed the importance of self-esteem in childhood for internalizing disorders in adolescence independent of these health behaviours. Examples of such programs include comprehensive school health programs. Their benefits to physical health have been demonstrated [30, 54], but benefits for mental health have yet to be examined and reported.

Strengths of this study include the use of a large population-based sample linked with longitudinal administrative health data, the use of a prospective design, the consideration of a variety of potential confounders including coinciding health behaviours, socio-demographic factors and self-esteem, the use of health care provider diagnoses for internalizing disorders which provides an objective and clinically meaningful assessment of internalizing disorders, and lastly, the fact that we monitored the outcome of interest for a period of 8 years, which is longer than in the few other studies. The interpretation of the study findings needs to consider several limitations. Assessment of child physical activity and sedentary behaviours used self-report, and thus is prone to recall bias. Food intake was also based on self-reported food frequency, which may also be subject to error, although the Harvard YAQ measure has been validated in this population [55]. Objective measures of these behaviours in future studies would allow for more accurate assessment for the exposure-outcome relations. Finally, in assessing internalizing disorders, we only considered children who had contacts with health-care providers relating to this diagnosis. Children with internalizing disorders who did not seek health care services or had barriers in accessing health care during the follow-up period may have been missed. This may have resulted in an underestimation of both the numbers of children experiencing internalizing disorders and the number of health care visits.

## Conclusions

We revealed in this study that variety in the diet, physical inactivity and sedentary behaviour in childhood are independently associated with the development of internalizing disorders in adolescence. These findings suggest that health promotion programs aiming to improve children’s diets and physical activity behaviours may also contribute to the prevention of mental disorders, providing further evidence that health behaviours and mental health are linked.

## Additional file

Additional file 1: Table S1. Logistic regressions for the associations of health behaviours in childhood with internalizing disorder in adolescence among participants of the Children’s Lifestyle and School Performance Study, Nova Scotia, Canada. (DOCX 20 kb)

## Abbreviations

CIHI: Canadian Institute for Health Information
CLASS: Children’s Lifestyle and School Performance Study
DAD: Discharge Abstract Database
DQI-I: Diet quality index-international
ICD-10-CA: International Classification of Diseases, Tenth Revision, Canadian version
ICD-9-CM: International Classification of Diseases, Ninth Revision, Clinical Modification
MSI: Medical Services Insurance
NBM: Negative binomial regression models
PA: Physical activity
