# Supplementary material for: The importance of health behaviours in childhood for the development of internalizing disorders during adolescence
Source: BMC Psychol. 2017 Dec 12;5:38. doi: 10.1186/s40359-017-0208-x (PMC5727924; doi:10.1186/s40359-017-0208-x)
Supplement: Additional file 1: Table S1. — Logistic regressions for the associations of health behaviours in childhood with internalizing disorder in adolescence among participants of the Children’s Lifestyle and School Performance Study, Nova Scotia, Canada. (DOCX 20 kb) [file 40359_2017_208_MOESM1_ESM.docx]

**Supporting information**

**Table S1 Logistic regressions for the associations of health behaviours in childhood with internalizing disorder in adolescence among participants of the Children’s Lifestyle and School Performance Study, Nova Scotia, Canada**

| **Variable** | **Univariate model** | **Multivariable model** |
| --- | --- | --- |
|  | **OR (95% CI)** | **OR (95% CI)** |
| **DQI-I variety** (Reference: Lowest tertile) |  |  |
| Middle tertile | 0.90 (0.77, 1.05) | 0.90 (0.73, 1.11) |
| Highest tertile | 0.91 (0.77, 1.08) | 0.91 (0.68, 1.21) |
| **DQI-I adequacy** (Reference: Lowest tertile) |  |  |
| Middle tertile | 1.04 (0.88, 1.22) | 1.16 (0.92, 1.48) |
| Highest tertile | 0.97 (0.82, 1.14) | 1.03 (0.74, 1.43) |
| **DQI-I moderation** (Reference: Lowest tertile) |  |  |
| Middle tertile | 0.93 (0.80, 1.07) | 0.95 (0.77, 1.17) |
| Highest tertile | **1.23 (1.02, 1.48)** | 1.31 (0.98, 1.74) |
| **DQI-I balance** (Reference: Poor balance (score<1) |  |  |
| Good balance (score≥1) | 1.11 (0.96, 1.27) | 1.03 (0.85,1.24) |
| **DQI-I overall** (Reference: Lowest tertile) |  |  |
| Middle tertile | 0.89 (0.75, 1.05) | 0.95 (0.76, 1.19) |
| Highest tertile | 1.01 (0.85, 1.18) | 1.03 (0.76, 1.41) |
| **Physical activity without coach** (Reference: Never) |  |  |
| 1 to 3 times/week | 0.79 (0.63, 1.01) | 0.81 (0.62, 1.05) |
| ≥4 times/week | **0.78 (0.62, 0.98)** | 0.85 (0.66, 1.09) |
| **Physical activity with coach** (Reference: Never) |  |  |
| 1 to 3 times a week | **0.83 (0.72, 0.96)** | 0.95 (0.80, 1.12) |
| ≥4 times/week | **0.73 (0.60, 0.89)** | 0.83 (0.67, 1.03) |
| **Use computer or play video games** (Reference: <1 hour/day) |  |  |
| 1-2 hours/day | **1.21 (1.04, 1.40)** | **1.31 (1.11, 1.54)** |
| 3-4 hours/day | 1.20 (0.96, 1.49) | 1.26 (0.99, 1.62) |
| ≥5 hours/day | 1.06 (0.79, 1.42) | 1.19 (0.84, 1.67) |
| **Watch TV**(Reference: <1 hour/day) |  |  |
| 1-2 hours/day | 0.84 (0.69, 1.03) | 0.89 (0.72, 1.11) |
| 3-4 hours/day | 0.94 (0.76, 1.16) | 0.93 (0.74, 1.18) |
| ≥5 hours/day | 0.93 (0.73, 1.18) | 0.92 (0.70, 1.22) |
| **Body weight** (Reference: Normal weight) |  |  |
| Overweight | 0.98 (0.82, 1.17) | 0.97 (0.81, 1.18) |
| Obese | 0.88 (0.68, 1.14) | **0.74 (0.56, 0.98)** |
| **Gender** (Reference: Boys) |  |  |
| Girls | **1.58 (1.38, 1.81)** | **1.62 (1.39, 1.89)** |
| **Residency** (Reference: Rural) |  |  |
| Urban | **1.24 (1.07, 1.43)** | **1.36 (1.16, 1.60)** |
| **Parental education** (Reference: Secondary school or less) |  |  |
| College | 0.89 (0.75, 1.05) | 0.93 (0.77, 1.11) |
| University or above | **0.78 (0.66, 0.94)** | 0.99 (0.80, 1.22) |
| **Household income** (Reference: ≤$20,000) |  |  |
| $20,001 - $40,000 | **0.70 (0.54, 0.91)** | **0.72 (0.54, 0.96)** |
| $40,001 - $60,000 | **0.63 (0.49, 0.82)** | **0.68 (0.51, 0.90)** |
| >$60,000 | **0.49 (0.38, 0.63)** | **0.52 (0.39, 0.69)** |
| **Self-esteem** (Low self-esteem vs. high self-esteem) |  |  |
| Self-perception | **1.38 (1.20, 1.58)** | **1.19 (1.02, 1.38)** |
| Externalizing problems | **1.20 (1.04, 1.38)** | 1.15 (0.98, 1.34) |
| Internalizing problems | **1.51 (1.31, 1.73)** | **1.34 (1.15, 1.55)** |
| Social-perception | **1.41 (1.23, 1.62)** | **1.21 (1.04, 1.41)** |

OR: odds ratio; 95% CI: 95% confidence interval; ORs in the multivariable model are mutually adjusted for all variables in the table, and for energy intake. Estimates are weighted to represent grade five students in Nova Scotia. Bold values for ORs and 95% CIs indicate statistical significance (p<0.05).
